# The clinical significance of serum HMGB1 in patients with lower extremity arteriosclerosis obliterans after interventional vascular restenosis

**DOI:** 10.3389/fsurg.2022.1031108

**Published:** 2023-01-06

**Authors:** Bo Yang, Zhang Xiaping

**Affiliations:** ^1^ Department of Clinical Nursing Teaching and Research Section, The Second Xiangya Hospital, Central South University, Changsha, China; ^2^ Department of Vascular Surgery, The Second Xiangya Hospital, Central South University, Changsha, China

**Keywords:** lower extremity arteriosclerosis obliterans, vascular interventional therapy, vascular restenosis, HMGB1, nomogram model

## Abstract

**Objective:**

This study explored the correlation between serum HMGB1 levels and postoperative vascular restenosis in patients with lower extremity arteriosclerosis obliterans (LEASO).

**Methods:**

A total of 362 patients LEASO who received vascular intervention were recruited in this study. Serum HMGB1 levels were measured by enzyme-linked immunosorbent assay. Logistic regression analysis was used to identify the influencing factors associated with vascular restenosis. The R procedure was used to create nomogram model. Receiver operating characteristic (ROC) analysis was used to determine the predictive value of serum HMGB1 and nomogram model for vascular restenosis.

**Results:**

Of the 362 LEASO patients included, 103 (28.45%) developed restenosis within 6 months of postoperative follow-up. Postoperative HMGB1 levels were significantly higher in patients with restenosis compared to those with non-restenosis. Postoperative HMGB1 levels were significantly and positively correlated with the severity of postoperative restenosis (*r *= 0.819). The AUC of postoperative HMGB1 for the diagnosis of postoperative restenosis was 0.758 (95% CI: 0.703–0.812), with a sensitivity and specificity of 56.31% and 82.24%, respectively. Multivariate logistic regression analysis showed that diabetes, smoking, regular postoperative medication, increased fibrinogen, decreased red blood cells, increased hs-CRP, and increased postoperative HMGB1 were independently associated with postoperative restenosis in patients with LEASO. The C-index of the nomogram prediction model constructed based on the seven influencing factors mentioned above was 0.918. The nomogram model was significantly more predictive of postoperative restenosis in LEASO patients compared with a single postoperative HMGB1 (AUC: 0.918, 95% CI: 0.757–0.934).

**Conclusion:**

Postoperative serum HMGB1 is an independent risk factor associated with postoperative vascular restenosis in patients with LEASO, and a novel nomogram model based on postoperative serum HMGB1 combined with clinical characteristics may help to accurately predict the risk of postoperative restenosis in patients with LEASO.

## Introduction

Lower extremity arteriosclerosis obliterans (LEASO) is a common disease in clinical vascular surgery, mostly in middle-aged and elderly people (1). 30% of LEASO occurs in iliac artery, and the other 70% are more common in the femoral artery, popliteal artery, and distal artery (2). The clinical manifestations of arteriosclerosis occlusion of the lower extremities often show intermittent claudication, resting pain, and gangrene; and the severe progress of which may even affect the life safety of the patient (3). The treatment methods of LEASO include vascular interventional therapy and vascular bypass surgery (4). Due to the large surgical trauma, minimally invasive interventional therapy, also known as percutaneous transluminal angioplasty (PTA), is often used, including balloon dilation and stent implantation. However, restenosis after interventional treatment has become a difficult and hot topic of clinical treatment. It was found that the restenosis rate in LEASO patients was 30%–50% within 6 months after intervention, and the incidence of restenosis within 1 year after intervention was as high as 70% (5).

There are many causes of restenosis after LEASO intervention, which are generally considered to be related to vascular inflammation, endothelial cell injury, and excessive proliferation and migration of smooth muscle cells (6). A large number of released inflammatory factors cause the body to show a high inflammatory state, which will aggravate the damage to vascular function and more easily lead to the occurrence of postoperative restenosis in patients. Neointimal proliferation is thought to underlie the pathophysiological development of restenosis, triggered by the pro-inflammatory molecules released due to endothelial damage, particularly during the thrombogenic and proliferative phases of restenosis (2). However, the mechanisms by which restenosis occurs after interventional procedures in patients with LEASO are still poorly understood. The search for reliable biomarkers to predict the occurrence of postoperative restenosis is of great importance.

High mobility group box protein B1 (HMGB1) is a key stress signal currently located in the nucleus with functions such as involvement in DNA transcription, induction of inflammatory responses, and regulation of vascular endothelial cell function (7). Studies have shown that HMGB1 is involved in the pathogenesis of atherosclerotic diseases. HMGB1 can be released from various cell types of atherosclerotic plaques, including smooth muscle cells, endothelial cells, and macrophages (8). Once released, HMGB1 exerts a variety of inflammatory effects on these cells. The pro-inflammatory effect of HMGB1 is mainly due to its structural characteristics and derived cellular biological effects (9). A variety of receptors play a role in HMGB1 signaling, including receptors for advanced non-enzymatic glycation end products (RAGEs) and members of the Toll-like receptors (TLRs) (10). TLRs bind to RAGEs to amplify the inflammatory response, an important mechanism that promotes atherosclerosis (11), and HMGB1 is the only known receptor with the highest affinity. Additionally, HMGB1 is also considered to be a neoendothelial mediator that stimulates vascular smooth muscle cell proliferation and migration (12). These data suggest that HMGB1 may be involved in the pathological process of restenosis after intervention. However, to date, studies on the relationship between circulating HMGB1 and restenosis after intervention in patients with LEASO remain unavailable.

In this study, we investigated the relationship between serum HMGB1 levels and postoperative vascular restenosis in 362 patients with LEASO who underwent vascular intervention. We also screened for risk factors associated with postoperative vascular restenosis based on Logistic regression analysis. Additionally, based on the results of logistic regression analysis, we developed a nomogram model combining serum HMGB1 with clinical characteristics to predict postoperative vascular restenosis in patients with LEASO. Our results suggested that serum HMGB1 levels may be a valuable biomarker for predicting restenosis after LEASO, which may provide insights for better understanding of the mechanisms of vascular restenosis.

## Materials and methods

### Objects

This study was approved by the ethics committees of The Second Xiangya Hospital, Central South University was performed in accordance with the Declaration of Helsinki. Written informed consent was obtained from all patients. LEASO patients hospitalized from 2016 to 2021 were collected. All patients met the following criteria: patients satisfied the diagnostic criteria for arteriosclerosis obliterans of the lower extremities and were confirmed to have superficial femoral artery disease, and lower extremity arterial intervention was performed; patients with Rutherford III–VI, manifesting as intermittent claudication, rest pain, tissue ulcers and gangrene; patients aged 40–80 years; patients with ankle brachial index (ABI) < 0.9; patients with regularly lower extremity arterial ultrasound or CT angiography after operation; patients not using ARBs, β receptor blockers or angiotensin-converting enzyme inhibitors ACEIs. The following patients were excluded: patients with a history of lower extremity trauma or surgery; patients with amputation or death after surgery; patients with severe hepatic or renal insufficiency; patients with residual stenosis probability >30% after surgery; patients who need traditional bypass surgery; patients with malignant tumors, autoimmune diseases, mental diseases, and acute and chronic inflammatory diseases.

### Interventional therapy

Femoral artery puncture was performed on the patient under local anesthesia. Then angiography of the lower extremity arteries was performed. Depending on the results of lower extremity computed tomography angiography (CTA), different interventional treatment options were adopted. If the stenosis of the vessel was greater than 50%, the appropriate balloon was selected to dilate the stenotic vessel; if the residual stenosis after balloon dilatation was greater than 30% (or endothelial stripping), endovascular stenting was performed after balloon dilatation of the stenotic vessel. The infrapopliteal artery was dilated using a 1.5–3.0 mm diameter, 120 mm length balloon (INV Atech) at a pressure of 8–14 atmospheres (1 atmosphere = 101.325 kPa) for 180 s, and a SMART self-expanding stent (Cordis, Miami Lakes, FL) was used in this study. After angiography, the luminal stenosis at the lesion site was less than 30%, and there was no significant arterial entrapment or serious complications related to the procedure, and the treatment was judged to be successful. The patient was given heparin 4000 U for 3 days; oral clopidogrel 75 mg/d for 1 month, followed by a long-term oral administration of aspirin 100 mg/d.

### Data collection

The basic information of the patients’ first admission was collected, including age, gender, course of disease, underlying diseases, smoking history, and drinking history.

The baseline biochemical indicators of patients before vascular interventional surgery were recorded, including systolic blood pressure (SBP), diastolic blood pressure (DBP), fasting blood glucose (FBG), high-sensitivity C-reactive protein (hs-CRP), total cholesterol (TC), triacylglycerol (TG), high density lipoprotein cholesterol (H-DLC), low density lipoprotein cholesterol (L-DLC), red blood cell count, white blood cell count, hemoglobin, and fibrinogen. Fasting venous blood was collected from patients before and 12 h after intervention. The level of HMGB1 was detected using a commercial human HMGB1 ELISA kit (Beyotime, Shanghai, China).

### Follow-up and restenosis criteria

Patients were followed up monthly for 6 months. CTA or ultrasound examination should be performed at least once a month during the 6 months of follow-up. Criteria for restenosis after interventional therapy: CTA and color Doppler ultrasound of the lower extremity arteries showed that the degree of vascular stenosis was >50%. The severity of restenosis of each patient was calculated according to the following formula (15): severity (%) = (B-A)/A × 100%, where A was the minimum lumen area at the stenosis area after interventional therapy and B was the minimum lumen area at the stenosis area at the end of the 6th month after interventional therapy. For patients with multiple vascular stenoses, the mean value was adopted to represent the severity of restenosis. The severity of restenosis was divided into four classes: I, 0%–20%; II, 20%–40%; III, >40%–60%; IV, >60%.

### Statistical analysis

The Kolmogorov-Smimov test was used to test whether the measurement data conformed to a normal distribution. The normally distributed measurement data were expressed as mean centerSD, and the independent samples *t* test was performed in parallel. Non-normally distributed measurement data were expressed as medians (quartiles), and the Mann-Whitney *U* test was performed. The enumeration data were analyzed by the 2 test. Based on independent risk factors in multivariate Logistic regression analysis. R 4.2.1 (http://www.r-project.org) was used to perform all the graphics based on R packages “rms”, “ggplot2”, “pROC”, and “car”. Program on R package was provided as Supplementary file. The performance of the nomogram was evaluated by the Harrell Concordance Index (C-index). The receiver operating characteristic curve (ROC) was used to evaluate the predictive value of HMGB1 level and nomogram model for postoperative restenosis, and the results were expressed as the area under the curve (AUC) and the 95% confidence interval (95% CI) of the area. Generally, the diagnostic accuracy with an AUC equal or above 0.6 is considered acceptable (16). *P* < 0.05 was considered to represent a statistically significant difference.

## Results

### Baseline characteristics

A total of 362 patients with LEASO, including 103 patients (28.45%) with postoperative restenosis, were included in this study. Compared with the non-Restenosis group, the proportion of patients with postoperative restenosis who had diabetes, smoking history and irregular postoperative medication significantly increased, and the levels of FBG, TC, fibrinogen and hs-CRP were significantly increased, while the level of red blood cell count was significantly lower (Table 1).

**Table 1:** Baseline characteristics of LEASO patients.

Characteristics		Non-Restenosis	Restenosis		
Gender (Male)		136	56	0.102	0.749
Age (years)		59.94 ± 8.60	61.36 ± 9.168	1.388	0.166
BMI (kg/m2)		25.27 ± 3.13	25.75 ± 2.86	1.351	0.178
Course of disease (years)		4.52 ± 0.993	4.51 ± 1.11	0.056	0.956
Hyperlipidemia		152	66	0.894	0.344
Coronary heart disease		171	67	0.031	0.86
Stroke		138	47	1.726	0.189
Hypertension		167	67	0.01	0.919
Diabetes		113	71	18.877	<0.001
Smoking		117	77	25.932	<0.001
Drinking		132	46	1.172	0.279
Regular medication after surgery		176	33	38.954	<0.001
Rutherford	III–IV	145	57	0.012	0.911
	V–VI	114	46		
Bilateral lesions		39	11	1.187	0.276
Surgical approach	Balloon dilatation alone	92	38	0.06	0.806
	Balloon expansion + Stent implantation	167	65		
FBG (mmol/L)		7.77 ± 1.00	8.02 ± 1.01	2.205	0.028
TC (mmol/L)		4.66 ± 1.05	5.13 ± 0.99	4.441	<0.001
TG (mmol/L)		1.51 ± 0.75	1.53 ± 0.72	0.225	0.822
L-DLC (mmol/L)		1.50 ± 0.83	1.43 ± 0.72	0.756	0.45
H-DLC (mmol/L)		1.10 ± 0.49	1.12 ± 0.53	0.177	0.859
SBP (mmHg)		140.23 ± 18.71	139.63 ± 17.75	0.417	0.677
DBP (mmHg)		84.47 ± 7.05	84.43 ± 7.21	0.044	0.965
Fibrinogen (g/L)		3.43 ± 0.93	4.03 ± 0.96	5.562	<0.001
Hemoglobin (g/L)		119.24 ± 19.73	117.71 ± 18.57	0.68	0.497
Platelet count (10^9^/L)		211.94 ± 79.22	199.26 ± 74.85	1.395	0.164
Red blood cells (10^9^/L)		4.19 ± 0.59	3.86 ± 0.55	4.72	<0.001
WBC (109/L)		7.69 ± 2.02	7.94 ± 1.94	1.076	0.283
hs-CRP (mg/L)		1.98 ± 0.49	2.27 ± 0.60	4.72	<0.001
Preoperative HMGB1 (pg/ml)		85.15 ± 6.81	84.21 ± 6.03	1.23	0.219
Postoperative HMGB1 (pg/ml)		42.44 ± 10.09	53.78 ± 12.07	9.107	<0.001

### Correlation between serum HMGB1 level and restenosis in LEASO patients

**Figure 1:**
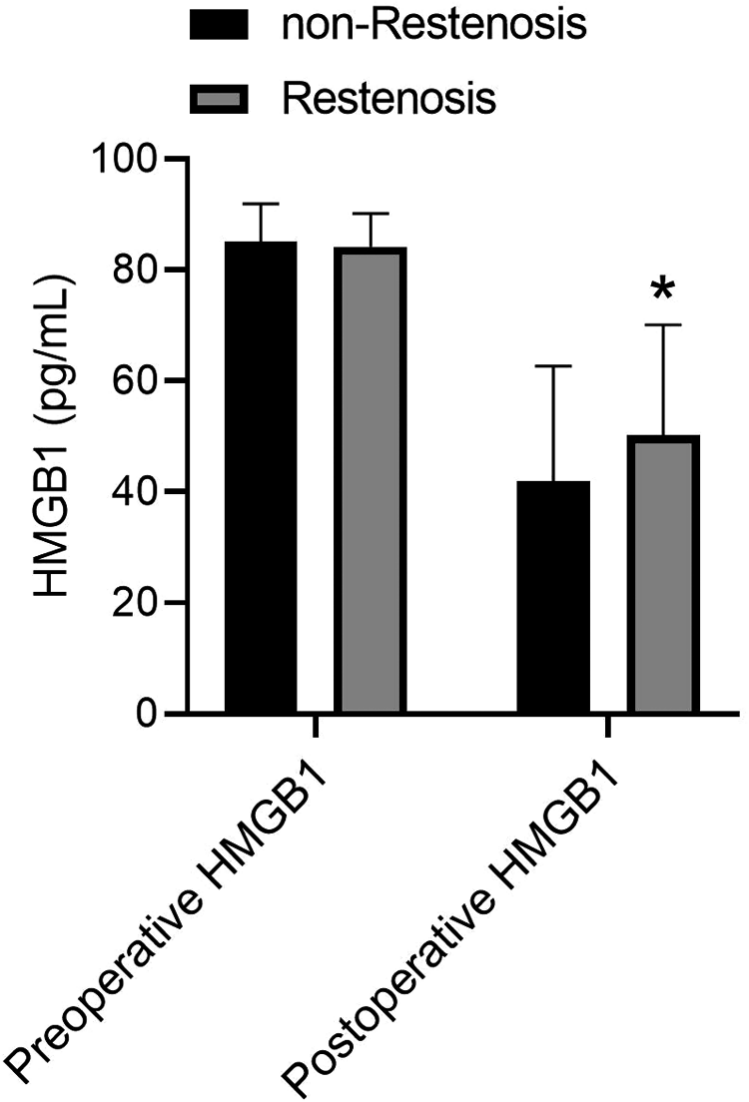
Comparison of serum HMGB1 levels between restenosis group and non-restenosis group.

**Figure 2:**
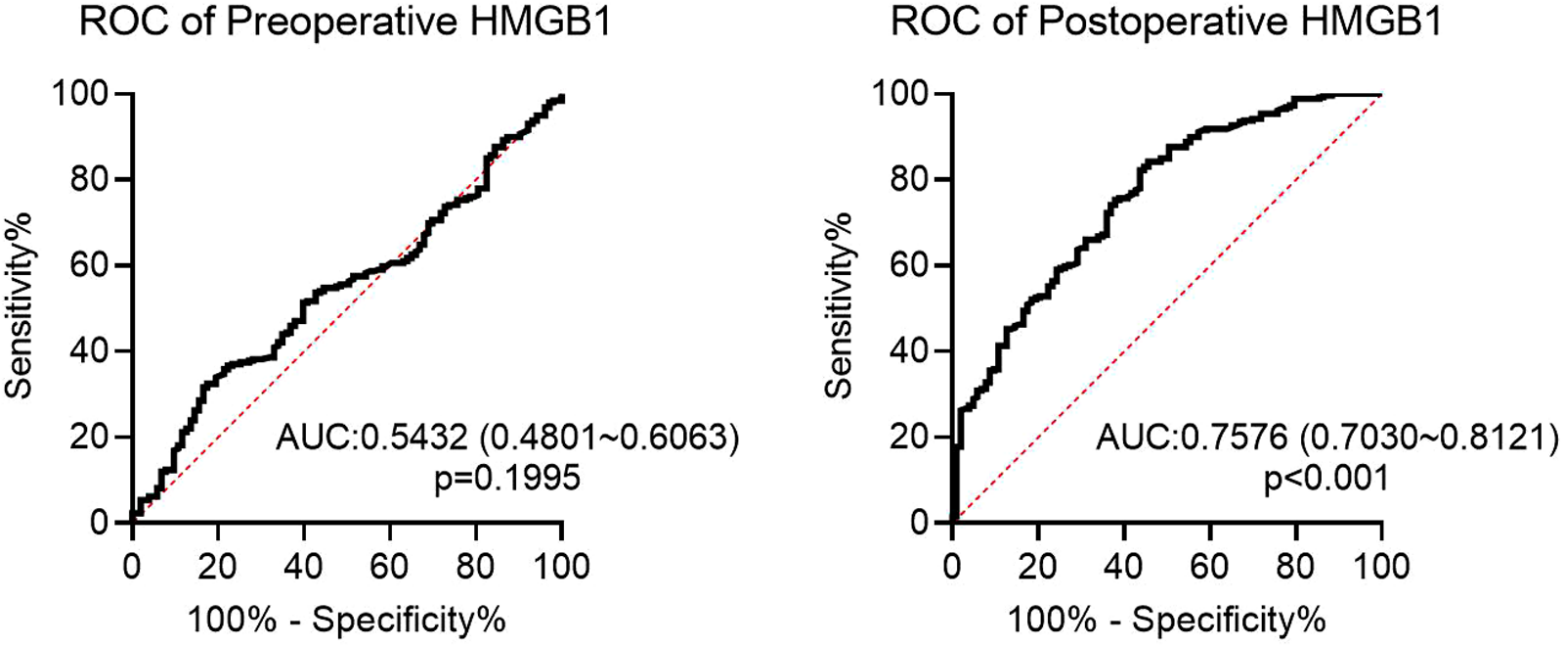
ROC curve of preoperative or postoperative HMGB1 for diagnosis of restenosis in LEASO patients.

Subsequently, preoperative and postoperative serum HMGB1 levels were compared between Restenosis group and non-Restenosis group. There was no significant difference in preoperative HMGB1 levels between the two groups (Figure 1). The postoperative HMGB1 level in the Restenosis group was significantly higher than that in the non-Restenosis group (Figure 1). Spearman correlation analysis showed that postoperative HMGB1 levels were significantly and positively correlated with the severity of postoperative restenosis (Table 2). Subsequently, the efficacy of preoperative and postoperative serum HMGB1 in the diagnosis of postoperative restenosis in LEASO patients was evaluated by ROC (Figure 2). The AUC of postoperative HMGB1 for the diagnosis of postoperative restenosis was 0.758 (95%CI: 0.703–0.812), and the sensitivity and specificity were 56.31% and 82.24%, respectively.

**Table 2:** Association between serum HMGB1 levels and the severity of restenosis in LEASO patients.

	I (0%–20%) (*n* = 59)	II (20%–40%) (*n* = 20)	III (40%–60%) (*n* = 13)	IV (>60%) (*n* = 11)	r	*p*
Preoperative HMGB1 (ng/ml)	83.44 ± 6.00	85.57 ± 6.93	84.04 ± 5.09	86.05 ± 5.41	0.131	0.188
Postoperative HMGB1 (ng/ml)	46.45 ± 7.27	56.33 ± 7.65	65.02 ± 6.23	75.14 ± 4.92	0.819	<0.001

### Logistic regression analysis of the influencing factors of postoperative restenosis in patients with LEASO

The risk factors with significant statistical significance in univariate regression analysis were included in multivariate Logistic regression. The results showed that diabetes, smoking, increased fibrinogen, decreased red blood cells, elevated hs-CRP, and increased postoperative HMGB1 were independent risk factors for postoperative restenosis in patients with LEASO, while regular postoperative medication was a protective risk factor (Table 3).

**Table 3:** Logistic regression analysis for risk factors for postoperative restenosis in LEASO patients.

Factors	*β*	S.E.	Wald	OR	95%CI	*p*
Diabetes	0.805	0.346	5.412	2.237	1.135–4.410	0.020
Smoking	2.077	0.393	27.940	7.984	3.696–17.249	<0.001
Regular medicine	−2.479	0.389	40.600	0.084	0.039–0.180	<0.001
Fibrinogen	0.792	0.201	15.571	2.209	1.490–3.274	<0.001
Red blood cells	−0.798	0.301	7.038	0.450	0.250–0.812	0.008
hsCRP	0.865	0.328	6.949	2.376	1.248–4.521	0.008
Postoperative HMGB1	0.121	0.019	41.039	1.128	1.087–1.171	<0.001

### Establishment and validation of predictive nomogram model

**Figure 3:**
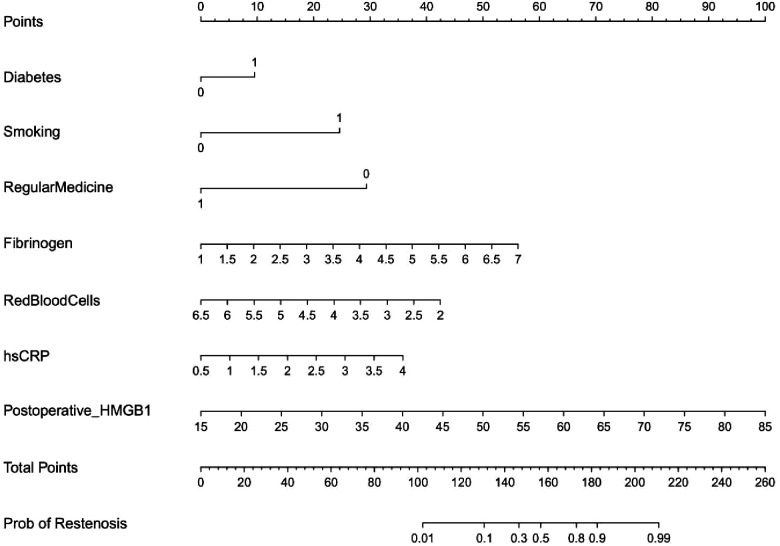
Nomogram for predicting vascular restenosis after intervention in LEASO patients.

**Figure 4:**
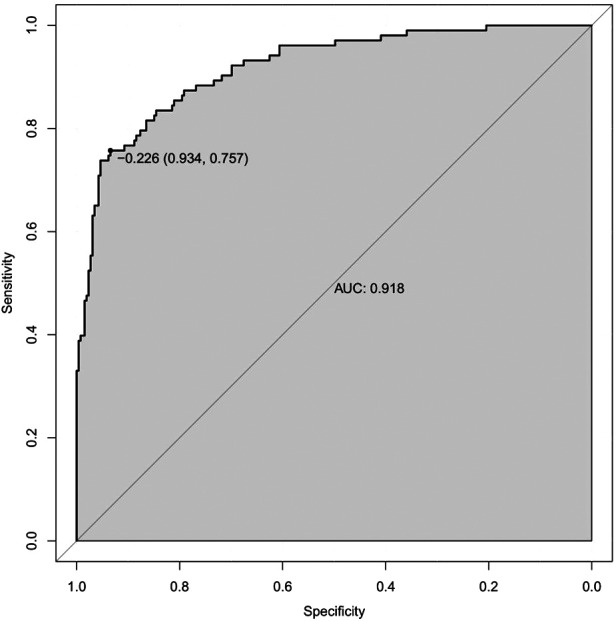
ROC curve used to verify the discriminative ability of the nomogram.

**Figure 5:**
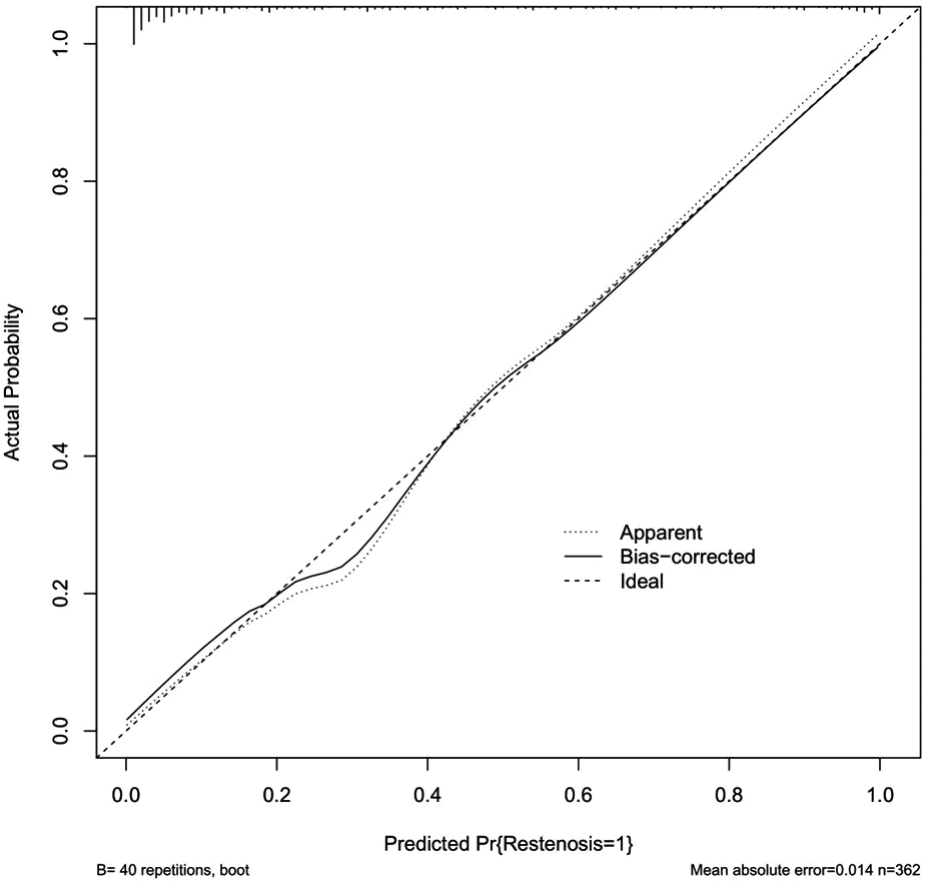
Calibration map of post-interventional vascular restenosis in LEASO patients.

We incorporated the seven independent risk factors mentioned above in an individualized nomogram prediction model for restenosis risk based on Logistic regression analysis (Figure 3). The nomogram model was evaluated by Harrell concordance index and ROC curve. Internal validation showed that the nomogram could accurately predict the C-index of restenosis, which was 0.918. A ROC curve was generated to verify the predictive accuracy of the nomogram, with an AUC of 0.918 (95%CI: 0.757–0.934; *P* < 0.001) (Figure 4). The consistency of the nomogram model for predicting postoperative restenosis in LEASO patients was evaluated by calibration curve. The results showed that the nomogram model predicted the probability of postoperative restenosis in LEASO patients was in good agreement with the actual probability (Figure 5).

## Discussion

Lower extremity arteries are one of the common arteries prone to atherosclerosis in addition to carotid and coronary arteries (13, 14). Vascular intervention has become the main treatment for LEASO because of its minimally invasive and significant efficacy (15, 16). It is well known that endovascular restenosis after interventional treatment is a difficult aspect of clinical treatment (17). A meta-analysis on the outcome of ischemic restenosis treatment of lower extremity arteries in patients with LEASO showed that the 3-year primary patency rate of stenting for long-segment iliac or superficial femoral artery occlusions was only 50% (18). It is important to explore biomarkers associated with the occurrence of restenosis. The present study demonstrates for the first time that elevated serum HMGB1 levels are a risk factor for restenosis after LEASO intervention. In addition, this study combined postoperative serum HMGB1 and clinical characteristics to construct a nomogram to predict the occurrence of restenosis after LEASO intervention.

In human atherosclerotic lesions from the aorta, carotid and coronary arteries, HMGB1 expression was significantly increased in the nucleus and cytoplasm of macrophages and smooth muscle cells located near the intima compared with normal human arteries (19). Strong expression of HMGB1 was also observed in regions adjacent to the necrotic core of atherosclerotic lesions (20). Intimal hyperplasia and restenosis following carotid balloon injury have been shown to be associated with enhanced HMGB1 expression (21, 22). Inhibition of HMGB1 can prolong scaffold life by regulating smooth muscle cell proliferation and neointima formation (23). Moreover, regulation of the inflammatory response by HMGB1 also plays a role in the response to stent injury (24, 25). The present study showed that postoperative serum HMGB1 levels were significantly higher in patients with restenosis than in patients without restenosis. However, there was no significant difference in preoperative serum HMGB1 levels between the two groups. Additionally, postoperative HMGB1 levels were positively correlated with the severity of postoperative restenosis. These data suggest that HMGB1 is involved in the occurrence of restenosis after the intervention. Higher serum HMGB1 levels are considered to be a potential marker of subclinical atherosclerosis (26). In the correlation study between Gensini score of coronary heart disease angiography and serum HMGB1 level, serum HMGB1 level was increased with disease progression, speculating that HMGB1 was a new inflammatory marker to predict the evolution of coronary stenosis degree in atherosclerotic heart disease (27). In the current study, ROC analysis showed that postoperative HMGB1 levels had a high predictive value for the occurrence of restenosis after interventional therapy in LEASO patients. Furthermore, Logistic regression analysis showed that postoperative serum HMGB1 level was an independent risk factor for restenosis in LEASO patients after interventional therapy. Collectively, postoperative serum HMGB1 can be used as a new potential serum biomarker for predicting restenosis after interventional treatment in patients with LEASO.

Timely detection and control of risk factors related to restenosis or even occlusion of blood vessels or lumen heads in postoperative patients can improve the postoperative prognosis of LEASO patients (16, 28). There are many factors that affect postoperative restenosis. Previous studies have shown that diabetes, hypertension, and smoking status are risk factors for postoperative restenosis in LEASO patients (29, 30). hs-CRP is a common indicator reflecting the inflammatory state (31), and its elevation has been confirmed to be a risk factor for recurrence after intervention (32). Fibrinogen is a new indicator of oxidative stress and inflammation in the body in recent years (33). A higher plasma fibrinogen concentration, as a separate variable, had a similar effect on cardiovascular risk as well-known risk factors such as smoking cigarette, obesity, arterial hypertension, and diabetes (34). In our study, diabetes, smoking, regular medicine, fibrinogen, red blood cells, hs-CRP were also confirmed to be independent risk factors for restenosis after interventional therapy in LEASO patients.

Predictor identification and risk assessment are critical for effective medical decision-making to prevent restenosis (35). The risk of major cardiovascular events and mortality in patients with diabetic nephropathy was significantly improved by multifactorial intervention (36). Therefore, treatment of relevant risk factors will help to reduce the incidence of restenosis in patients with LEASO undergoing interventional therapy. Nomogram-based predictive models have been widely used in clinical research (37). In the present study, the aforementioned independent risk factors were included in the prediction model we constructed with an optimal C-index of 0.918. Moreover, the nomogram-based prediction model was effective in predicting the occurrence of restenosis. Furthermore, the combination of six independent influencing factors associated with postoperative restenosis predicted the occurrence of restenosis more accurately than postoperative serum HMGB1 alone.

This study has several limitations. First, it was a single-center retrospective study with a small sample size. Second, the follow-up period of this study was short and the analysis of the influencing factors was not comprehensive enough. Because the mechanisms of restenosis after lower extremity arterial vascular intervention are very complex, it is necessary to expand the sample size, extend the follow-up time, and add different influencing factors (such as different drugs) to further explore the mechanisms in the future. In addition, the present study only analyzed the HMGB1 level at 12 h after intervention, and it is not yet possible to determine whether it is the best indicator for evaluation. The dynamic changes of HMGB1 still need to be observed in the follow-up, and the nomogram model still needs to be supported by data from the validation group.

In conclusion, this study demonstrates for the first time that HMGB1 levels in postoperative serum samples from patients with LEASO are significantly elevated after intervention and that postoperative HMGB1 levels have a high predictive value for the development of restenosis. In addition, elevated postoperative serum HMGB1 levels were an independent risk factor for restenosis after interventional treatment. A nomogram model constructed from postoperative HMGB1 combined with clinical characteristics can effectively assess the occurrence of restenosis after intervention in patients with LEASO. HMGB1 may become a therapeutic target for revascularization.

## Data Availability

The raw data supporting the conclusions of this article will be made available by the authors, without undue reservation.
